# Laser-Induced Methanol Decomposition for Ultrafast Hydrogen Production

**DOI:** 10.34133/research.0132

**Published:** 2023-05-09

**Authors:** Weiwei Cao, Yinwu Li, Bo Yan, Zhiping Zeng, Pu Liu, Zhuofeng Ke, Guowei Yang

**Affiliations:** ^1^ State Key Laboratory of Optoelectronic Materials and Technologies, Nanotechnology Research Center, Sun Yat-sen University, Guangzhou 510275, P. R. China.; ^2^ School of Materials Science and Engineering, Sun Yat-sen University, Guangzhou 510275, P. R. China.

## Abstract

Methanol (CH_3_OH) is a liquid hydrogen (H_2_) source that effectively releases H_2_ and is convenient for transportation. Traditional thermocatalytic CH_3_OH reforming reaction is used to produce H_2_, but this process needs to undergo high reaction temperature (e.g., 200 °C) along with a catalyst and a large amount of carbon dioxide (CO_2_) emission. Although photocatalysis and photothermal catalysis under mild conditions are proposed to replace the traditional thermal catalysis to produce H_2_ from CH_3_OH, they still inevitably produce CO_2_ emissions that are detrimental to carbon neutrality. Here, we, for the first time, report an ultrafast and highly selective production of H_2_ without any catalysts and no CO_2_ emission from CH_3_OH by laser bubbling in liquid (LBL) at room temperature and atmospheric pressure. We demonstrate that a super high H_2_ yield rate of 33.41 mmol·h^−1^ with 94.26% selectivity is achieved upon the laser-driven process. This yield is 3 orders of magnitude higher than the best value reported for photocatalytic and photothermal catalytic H_2_ production from CH_3_OH to date. The energy conversion efficiency of laser light to H_2_ and CO can be up to 8.5%. We also establish that the far from thermodynamic equilibrium state with high temperature inside the laser-induced bubble and the kinetic process of rapid quenching of bubbles play crucial roles in H_2_ production upon LBL. Thermodynamically, the high temperature induced using laser in bubbles ensures fast and efficient release of H_2_ from CH_3_OH decomposition. Kinetically, rapidly quenching of laser-induced bubbles can inhibit reverse reaction and can keep the products in the initial stage, which guarantees high selectivity. This study presents a laser-driven ultrafast and highly selective production of H_2_ from CH_3_OH under normal conditions beyond catalytic chemistry.

## Introduction

Hydrogen (H_2_) energy, the cleanest energy source with a high energy density, is one of the foundations of the future energy system [1–3]. However, H_2_ is flammable and explosive, as well as inconvenient for transport [4,5]. Therefore, in situ H_2_ production is an effective way to solve this problem. Methanol (CH_3_OH) is a promising liquid H_2_ carrier and is widely used for H_2_ production [6,7]. Thermocatalytic CH_3_OH reforming is a traditional technique of releasing H_2_ from CH_3_OH, but this process needs to be carried out at relatively high temperatures (usually 200 °C) and, at the same time, a large amount of carbon dioxide (CO_2_) is emitted [8–12]. Therefore, scientists have been looking for mild and sustainable ways to produce H_2_ from CH_3_OH. Photocatalysis and photothermal catalysis carried out at a normal temperature have attracted extensive attention [12–16]. However, for these methods above, to avoid carbon monoxide (CO) poisoning of a catalyst, CO_2_ emission is inevitable [17–19]. This should be detrimental to carbon neutrality and exacerbate a greenhouse effect. In fact, CO can be used as fuel [20]. Thus, how to realize the efficient and rapid release of H_2_ from CH_3_OH without CO_2_ emission under mild conditions is indeed a challenge.

**Fig. 1.**
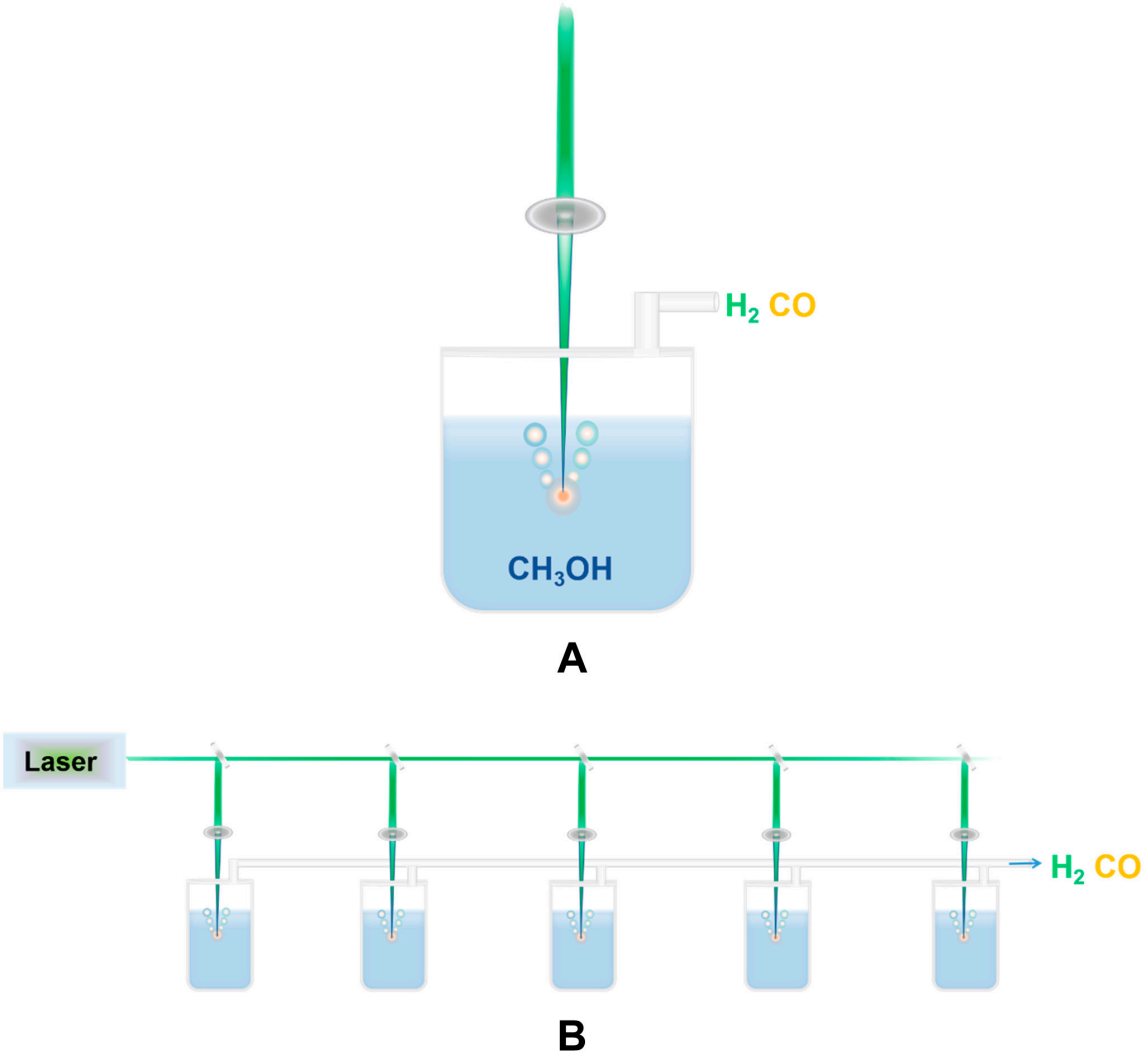
Schematic illustration of the experiment set. (A) A second harmonic is produced using a Q-switched Nd:YAG laser with a pulse wavelength of 532 nm and a pulse width of 10 ns. A pulsed laser is focused inside the liquid, forming a spot size of about 1 mm. Finally, the gas produced by the reaction is collected. (B) A laser-driven amplification scheme of H_2_ release from CH_3_OH is proposed, which divides the beam into multiple beams of light, forms an array, and expands the scale of production.

Here, we, for the first time, report an ultrafast and highly selective production of H_2_ without any catalysts and no CO_2_ emission from CH_3_OH by laser bubbling in liquid (LBL) at a normal temperature and pressure. LBL means that the pulsed laser is focused below the liquid level to produce small bubbles in liquid as shown in Fig. 1. Note that the temperature inside the laser-induced bubble can reach as high as about 10^4^ K [21,22]. At such high temperatures, the ionization temperature of CH_3_OH has been exceeded, thus generating CH_3_, CH_3_O, and CH_2_OH radicals as well as CH_3_O^−^, OH^−^, and H^+^ ions in the bubble. At the same time, these bubbles would quench at a very short time because of the confining and cooling effect of the surrounding liquid, which would cause that the primary products of high-temperature chemical reactions occurring inside bubbles are directly frozen and retained at room temperature [23–25]. Our measurements show that, in such a local extreme environment inside bubbles, CH_3_OH is directly to release H_2_ (33.41 mmol·h^−1^) and CO (16.88 mmol·h^−1^). The production rate of H_2_ is much higher than that of photocatalytic and photothermal catalysis. Meanwhile, the selectivities for H_2_ and CO are up to 94.26% and 89.25%, respectively. At 700 mJ per pulse of laser energy, the conversion efficiency from laser light to H_2_ and CO can reach 8.5%. Notably, there is no CO_2_ emission. Additionally, we establish the mechanism of laser-driven CH_3_OH releasing H_2_, including the thermodynamics and kinetics of high-temperature reactions in the bubble upon the LBL process. In terms of thermodynamics, the extreme high temperature of the bubble caused using laser promotes the efficient reaction and ensures a high yield. From a kinetic point of view, the rapid quenching process of bubbles freezes the final product, and the high-energy barrier of the reverse reaction prevents the product from returning to CH_3_OH. Therefore, the synergistic effect of thermodynamics and kinetics ensures the high yield and selectivity of H_2_ release from CH_3_OH. Therefore, these results show that the laser-driven direct decomposition of CH_3_OH to release H_2_ has great potential in the industry.

## Results and Discussion

A schematic of the experimental setup is shown in Fig. 1, and the whole LBL process was carried out at room temperature and atmospheric pressure. In this case, LBL is used to enable rapidly switching reactions between high temperature and room temperature to achieve the release of H_2_ from CH_3_OH (Fig. 1A). When LBL is performed, the bubbles are generated instantaneously using the focused laser and then collapsed in several hundred microseconds. A series of high-temperature chemical reactions would occur before the collapse of bubbles, and then products would be released together with the collapse of bubbles. Note that after the experiment was completed, the solution temperature remained basically unchanged. Figure 1B shows our proposal for the laser-driven H_2_ production from CH_3_OH in practical applications. When the laser energy is excess, the laser beam can be divided to make the energy utilization rate higher. Dividing laser into multiple reaction devices to assemble them into an array can enhance the production rate for large-scale operations. Evidently, this technique opens a new direction for the industrial realization of H_2_ production from CH_3_OH.

**Fig. 2.**
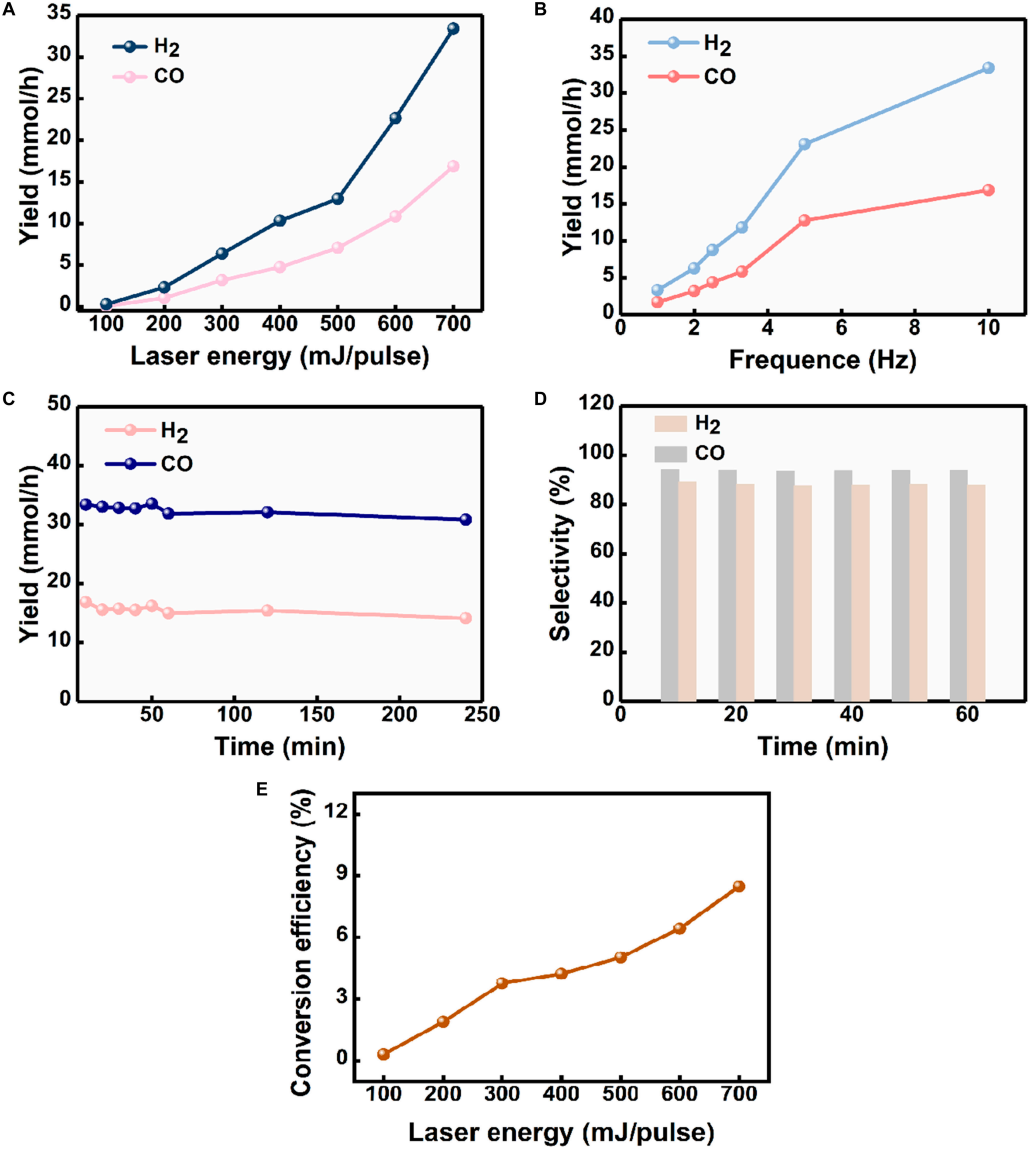
Measurement and characterization of the products. (A)The yield rate versus laser energy and (B) frequency of pulsed laser. (C) The yield rate and (D) selectivity of H_2_/CO at different times under 700 mJ per pulse of laser energy. (E) Laser-to-hydrogen conversion efficiency versus laser energy.

A series of measurements of the samples were performed as shown in Fig. 2. The yields of the generated gases positively correlated with the laser energy (Fig. 2A). The H_2_ yield is close to 0 when the laser energy is below 100 mJ per pulse and then increases with the increase of laser energy. Therefore, the 100 mJ per pulse is the threshold of laser energy for the reaction to occur. When the laser energy is higher than 100 mJ per pulse, the reaction can be carried out and a large amount of H_2_ can be produced. At 700 mJ per pulse, the yields of H_2_ and CO can reach 33.41 and 16.88 mmol·h^−1^, respectively. These results can be attributed to the increase in the temperature in bubbles caused by the increase in the laser energy, which leads to more efficient high-temperature chemical reactions. It is worth noting that the process has only trace amounts of CH_4_, C_2_H_4_, C_2_H_6_, and C_2_H_2_ generation, which have rates that were only 0.56, 0.22, 0.073, and 1.18 mmol·h^−1^ (Fig. S1), respectively. More importantly, there is no CO_2_ generation in the LBL process. In addition to adjusting the laser energy, we study the frequency effect of the pulsed laser. As can be seen from Fig. 2B, the maximum yield (33.41 mmol·h^−1^) of H_2_ was reached at the frequency of 10 Hz. However, only 3.34 mmol·h^−1^ of H_2_ and 1.76 mmol·h^−1^ of CO were measured at 1 Hz. Therefore, this result can be attributed to more bubble generation per unit of time with an increase in the frequency of pulsed laser. To investigate the effect of the reaction time on the yield rate, gas samples at various reaction times at 700 mJ per pulse of laser energy were collected to calculate the yield rate. As shown in Fig. 2C, the laser action time had no obvious effect on the yield rate at the same laser energy. This is mainly because the gas product is released as the bubble collapses, then the surrounding CH_3_OH solution will replenish this reaction area, and new bubbles will be generated again. Importantly, the laser-driven CH_3_OH decomposition releases H_2_ and CO with a high selectivity, up to 94.26% and 89.25%, respectively, as shown in Fig. 2D. Therefore, these results show that H_2_ yield does not change with laser action time and further elucidate that this method can sustainably release H_2_ from CH_3_OH over a long period of time through a laser-driven process. In addition, we also study the energy conversion efficiency from laser light to H_2_ and CO. Figure 2E shows that, with the increase of laser energy, the conversion efficiency is also higher, and the energy conversion efficiency can be as high as 8.5% at 700 mJ.

To evaluate advanced performance of LBL, we compared H_2_ yield rates of LBL with that of photocatalytic and photothermal catalytic H_2_ release from CH_3_OH as shown in Table. Considering the characteristic terms involved in photocatalytic and photothermal catalytic CH_3_OH releasing H_2_, such as solution and irradiation area, the yield rate was normalized into 1-mm space dimensions (e.g., irradiation area and the laser-focused spot). From Table, we can clearly see that the laser-driven H_2_ production from CH_3_OH is 3 orders of magnitude higher than the best value of photocatalysis and photothermal catalysis to date.

**Table.** Comparison of laser-driven H_2_ release from CH_3_OH with photocatalytic and photothermal catalytic H_2_ release from CH_3_OH at ambient temperature and pressure.

Normalized yield (mmol·h^−1^)	H_2_ production rate	Characteristic terms (irradiation area, catalyst loading, solution)	Reference
9.1 × 10^−4^	14 mmol·h^−1^·g^−1^ cat	3.84 cm^2^, 25 mg, MeOH/H_2_O (v/v = 1/1)	Cui et al. [25]
2.9 × 10^−2^	328 mmol·h^−1^·g^−1^ cat	8.5-mm diameter, 5 mg, CH_3_OH/H_2_O (v/v = 1/4)	Luo et al. [13]
6.6 × 10^−2^	310 mmol·h^−1^·g^−1^ cat	0.0471 m^2^, 10 g, CH_3_OH/H_2_O (v/v = 1/1.6)	Li et al. [12]
3.34 × 10^1^	33.4 mmol·h^−1^	1-mm diameter, catalyst-free, CH_3_OH	This study

Accordingly, these experimental results above suggest that CH_3_OH can efficiently release a large amount of H_2_ and CO upon the LBL process, and the yield rate can be flexibly regulated by changing the energy and frequency of the pulsed laser. Additionally, ultrafast and highly selective production of H_2_ and CO without CO_2_ emission from CH_3_OH can be achieved by LBL.

**Fig. 3.**
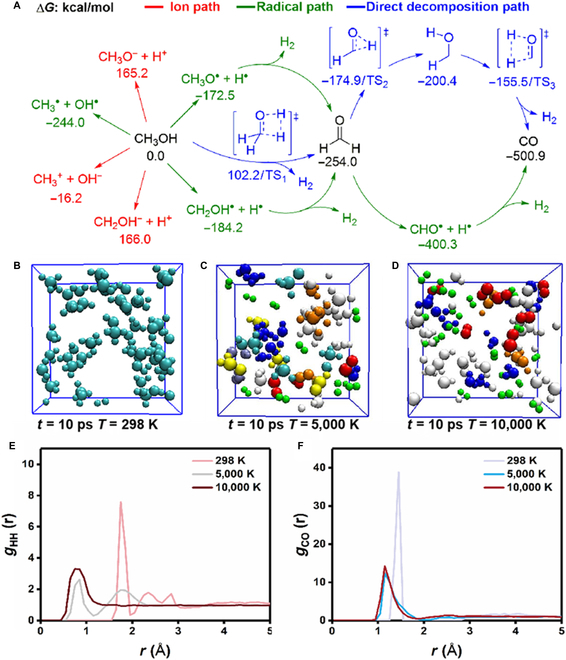
Theoretical calculations and simulations for CH_3_OH decomposition upon LBL. (A) Mechanism for CH_3_OH decomposition under the LBL process. The free energies calculated in 10,000 K. (B to D) Configurations of the nonadiabatic ab initio MD simulations. H_2_, CO, CH_3_OH/CH_3_O, H_2_O, CH_2_O, CH_4_/C_2_H_4_/C_2_H_2_, and other intermediates are shown in green, red, cyan, blue, yellow, orange, and white colors, respectively. (E and F) H–H and C–O radial distribution functions. The radial distribution functions are calculated for 500 fs for each temperature.

To have a deep understanding to CH_3_OH decomposition reaction under the LBL process, the theoretical research was taken on the basis of the density functional theory (DFT) calculation, aiming to explain the high-temperature chemical reactions occurring inside bubbles upon the LBL process. The pulsed laser would lead to the high-temperature environment, which promotes the CH_3_OH decomposition reaction. As shown in Fig. 3A, the 3 possible reaction paths are the (a) ion path, (b) radical path, and (c) direct decomposition path, respectively. In the ion paths, CH_3_OH can be decomposed into a series of ions species: CH_3_O^−^/H^+^ (165.2 kcal/mol), CH_2_OH^−^/H^+^ (166.0 kcal/mol), and CH_3_^+^/OH^−^ (−16.2 kcal/mol). The ions species are located at high-activation free energy, which are less favorable for the CH_3_OH decomposition, while the radical paths are more favorable for the CH_3_OH decomposition. As shown in Fig. S3, the formation of radical species requires lower temperature than that of ion species. When the reaction temperature is high than about 4,000 K, the CH_3_OH^•^/H^•^ and CH_2_OH^•^/H^•^ can form. The radical species CH_3_O^•^/H^•^ (−172.5 kcal/mol), CH_2_OH^•^/H^•^ (−184.2 kcal/mol), and CH_3_^•^/OH^•^ (−244.0 kcal/mol) are located at low-activation free energy. By releasing H_2_, both of CH_3_O^•^/H^•^ and CH_2_OH^•^/H^•^ species can form the key intermediate CH_2_O (−254.0 kcal/mol). The CH_2_O can be further decomposed via radical intermediate CHO^•^/H^•^ (−400.3 kcal/mol), resulting in the final product CO and H_2_ (−500.9 kcal/mol). In addition, the formation of C_2_H_6_, C_2_H_4_, C_2_H_2_, and CH_4_ are shown in Fig. S4, which are initial from the key radical species CH_3_^•^/OH^•^. The coupling of 2 CH_3_^•^ species would result in C_2_H_6_, which can form the C_2_H_4_ and C_2_H_2_ by releasing H_2_, while the coupling between CH_3_O^•^/H^•^ and CH_3_^•^/OH^•^ species would lead to CH_4_ and CH_2_O, which can generate CO by releasing H_2_. Furthermore, the direct decomposition paths have been studied as well. However, the transition states for the direct decomposition paths are located at high-activation free energy: TS_1_ (102.2 kcal/mol), TS_2_ (−174.9 kcal/mol), and TS_3_ (−155.5 kcal/mol).

Moreover, the ab initio molecular dynamics (MD) simulations were performed to explain the high-temperature chemical reaction of CH_3_OH in bubbles. The simulation temperatures are 298, 5,000, and 10,000 K, respectively, aiming to explore the temperature effect of pulsed laser with different energies. As the temperature rises from 298 to 5,000 K, the H_2_, CO, H_2_O, CH_2_O, and hydrocarbon products were observed in MD simulation (Fig. 3B and C). When the temperature rises to 10,000 K, a large amount of H_2_ and CO products were generated (Fig. 3D). Furthermore, the distributions of radial distribution functions (RDFs) exhibited the decomposition of the CH_3_OH. As the temperature rises to 10,000 K, the maximum distribution of hydrogen–hydrogen was shifted from 1.75 to 0.75 Å (Fig. 3E), while the maximum distribution of carbon–oxygen was changed from 1.45 to 1.25 Å (Fig. 3F). The hydrogen–hydrogen and carbon–oxygen RDFs show the formation of the H_2_ and CO molecules. In addition, the carbon–carbon RDF further suggested the formation of ethylene and acetylene (Fig. S5).

**Fig. 4.**
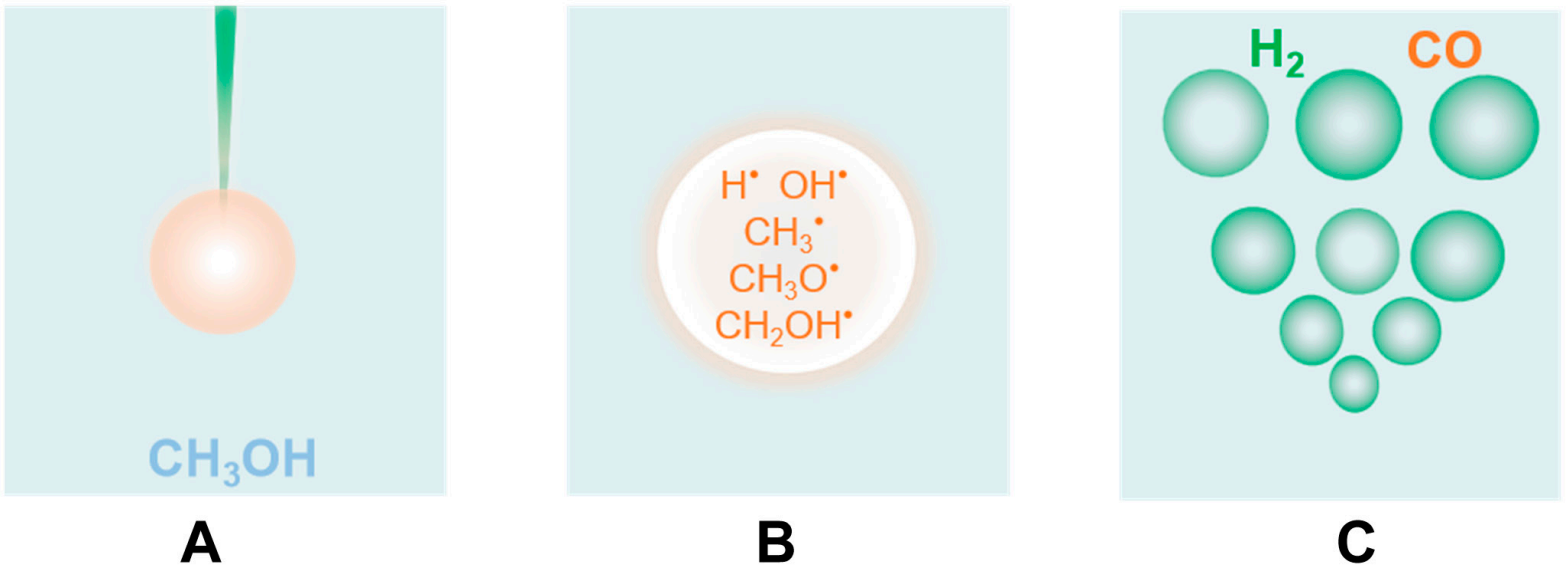
Mechanism of laser-driven H_2_ release from CH_3_OH. (A) A pulsed laser is focused below the solution surface to trigger bubbles. (B) The laser-induced bubble contains some high-energy ions from CH_3_OH, such as CH_3_^•^, H^•^, and CH_2_OH^•^. The bubble is a miniature reactor. (C) The products are released along with bubbles’ rapid collapsing.

To further understand the mechanism of laser-driven H_2_ release from CH_3_OH, we propose basic physical and chemical perspectives on the basis of the experimental measurements, DFT calculations, and MD simulations as shown in Fig. 4. First, when the pulsed laser is focused below the liquid level, many small bubbles form in the solution (Fig. 4A). A high-energy laser can induce high temperatures up to thousands of kelvin inside the bubble, and this extreme high temperature can exceed the ionization temperature of CH_3_OH in the bubble, which results in the formation of CH_3_, CH_3_O, and CH_2_OH radicals as well as CH_2_OH^−^, OH^−^, and H^+^ ions in the bubble (Fig. 4B). Note that the state in the bubble is far from thermodynamic equilibrium. Therefore, these laser-induced bubbles provide a favorable thermodynamic environment for the CH_3_OH decomposition.

On the basis of Eyring’s transition state theory, the temperature and the activation free energy are 2 key factors for the chemical reaction rate processes. By comparing with each reaction step in Fig. S6, the CH_3_OH decomposition is an exothermic reaction in the whole process. The reduction of activation free energy is mainly due to the entropy increase of the reactions. As shown in Fig. S6, the Δ*S* are 27.8 and 24.7 cal/mol for the formation of CH_2_O and CO reactions, respectively. In the LBL process, the bubble environment is located at the high temperature, which amplifies the entropy effect. Thus, these results show that the entropy effect plays an important role in the reaction process at a high temperature, which can directly and effectively promote the decomposition of CH_3_OH to release H_2_.

Kinetically, the pulsed laser results in an instantaneous formation of bubbles at the focal point inside of the CH_3_OH solution. According to previous studies, far from equilibrium bubbles would disintegrate within 100 to 400 μs [21], enabling ultrafast H_2_ release from CH_3_OH. Because the temperature drops to room temperature instantaneously, during the rapid cooling process, H_2_ and CO of the products formed by the high temperature can be retained, preventing the products from slowly decomposing and returning those to the initial state. Because of the rapid quenching of bubbles, the formation of free radical species is prevented. The activation free energy of the formation of CH_2_OH^•^/H^•^ is 86.6 kcal/mol in 298.15 K. In addition, the CO and H_2_ conversion reaction are carried out in 298.15 K as well (Fig. S7). The rate-determining step of CO conversion is the formation of CHOH, which undergoes the transition state TS_3_-iso. The activation free energy of this step is 81.0 kcal/mol. The temperature for a practical CO and H_2_ conversion reaction (*t*_1/2_ = 1 s to 1 h) is around 866.3 to 1038.9 K (Fig. S8). The results clearly suggested that the CO and H_2_ conversion are almost nonoccurrence below 1038.9 K. Additionally, the MD simulations were taken to simulate the rapid cooling stage of the LBL process. The RDFs results suggest that the H_2_ and CO species are retained in high proportion (Figs. S9 and S10). When the bubble induced using laser expands to the maximum volume in CH_3_OH, it is close to the vacuum state because the number of internal molecules does not change much. The external liquid pressure acts on the bubble, and the resulting pressure difference causes the bubble to collapse quickly. Therefore, rapid quenching of nonequilibrium ensures the formation of CO/H_2_ products in laser-induced high-temperature chemical reactions in bubbles, as well as prevented the products from further reversion to initial states. As a result, large amounts of H_2_ and CO are released as the bubble collapsed, and the process can achieve a high product yield (Fig. 4C).

Therefore, the LBL process can be carried out at room temperature and atmospheric pressure, which is simple and has no CO_2_ emissions. The nonequilibrium process of bubbles is similar to the microreactor induced using a laser, in which the high-temperature chemical reaction takes place, which can proceed the reaction more effectively. Moreover, the extremely short quenching time can keep the product at room temperature and inhibit the reverse reaction.

## Conclusion

In summary, we developed an ultrafast and highly selective production of H_2_ and CO without a catalyst as well as no CO_2_ emission from CH_3_OH driven using laser under normal temperature and pressure. A super high H_2_ yield rate of 33.41 mmol·h^−1^ with 94.26% selectivity can be produced upon the laser-driven process, and this yield is 3 orders of magnitude higher than the best value reported for photocatalytic and photothermal catalytic H_2_ production from CH_3_OH to date. On the basis of DFT calculation and MD simulation, we clarified the basic physical and chemical processes of laser-driven ultrafast H_2_ release from CH_3_OH, which confirm the experimental measurements of high temperature promoting CH_3_OH decomposition. We also establish that the thermodynamic and kinetic mechanism of high-temperature chemical reactions inside bubbles induced pulsed laser. Thermodynamically, the high temperature of bubbles amplifies the entropy effect during the CH_3_OH decomposition, which reduces the activation energy of the decomposition process without catalysts. Kinetically, the bubbles have a short quenching time, which makes it difficult for the products H_2_ and CO to return to their initial states through a reverse reaction. Therefore, this synergy between thermodynamics and kinetics results in high yield and selectivity for H_2_ and CO. Accordingly, we believe that LBL can be expected to be a disruptive technology to release H_2_ from CH_3_OH without CO_2_ emission at normal conditions beyond catalytic chemistry.

## Materials and Methods

### Synthesis and characterization

First, CH_3_OH (200 ml) was poured into a quartz reactor. Then, the second harmonics generated using a Q-switched Nd:YAG (neodymium-doped yttrium aluminum garnet crystal) laser device with a wavelength of 532 nm; a pulse width of 10 ns; repetition rates of 1, 5, and 10 Hz; and laser pulse energies of 100 to 700 mJ was focused inside the liquid, forming a spot size of about 1 mm. Moreover, not only the argon (Ar) gas is introduced into the solution, which can play the role of a protective gas, but also the gas produced during the reaction that flowed out together with the Ar. Before the experiment, the Ar gas was a bubble in CH_3_OH (0.4 l/min) for 60 min to ensure that no air was present in the water. Gases produced by the laser-driven CH_3_OH decomposition process were collected and characterized. The yields of gases (*y*_CO_, *y*_H_2__, *y*_C_2_H_2__, *y*_C_2_H_4__, *y*_CH_4__, and *y*_C_2_H_6__) under diverse laser energy and time were quantified by gas chromatography (GC-2014C, Shimadzu) with a thermal conductivity detector (5Å molecular sieve column) and 2 flame ionization detectors (MC-3 column). The formula for calculating H_2_ (*S*_H_2__) and CO (*S*_CO_) product selectivity was as follows:

$$S_{\mathrm{CO}}=\frac{y_{\mathrm{CO}}}{y_{\mathrm{CO}}+y_{C_{2}H_{2}}+y_{C_{2}H_{4}}+y_{{\mathrm{CH}}_{4}}+y_{C_{2}H_{6}}}\times 100\%$$

$$S_{H_{2}}=\frac{y_{H_{2}}}{y_{H_{2}}+y_{C_{2}H_{2}}+y_{C_{2}H_{4}}+y_{{\mathrm{CH}}_{4}}+y_{C_{2}H_{6}}}\times 100\%$$

Considering the enthalpy change of CH_3_OH decomposing to H_2_ and CO is as follows:

$$CH_{3}\mathrm{OH}⟶\mathrm{CO}+H_{2},∆H=128.07\;\mathrm{kJ}/\mathrm{mol}$$

The formula for calculating conversion of laser light to H_2_ and CO product selectivity was as follows:

$$\eta \;\left(\%\right)=\frac{\Delta H\times n\left(\text{methanol}\right)}{\text{laser energy}\;\left(\text{mJ}/\text{pulse}\right)\times 10\;\text{Hz}\times 3,600\;s/h}\times 100\%$$

where *η* is the energy conversion of laser light to H_2_ and CO, Δ*H* is the enthalpy change from CH_3_OH to CO and H_2_, and *n*(methanol) is the amount of CH_3_OH that takes part in reaction.

### Calculation and simulation

The mechanism study was taken by DFT calculation, aiming to explore the reaction path of the H_2_ release of CH_3_OH driven by LBL. The ab initio MD simulations were taken to explore the decomposition of CH_3_OH. The reaction rate and half-life are calculated on the basis of Eyring’s transition state theory. The details of all the calculation processes and equations are available in the Supplementary Materials.

## Data Availability

The data that support the findings of this study are available within the article and the Supplementary Materials. Raw data are available from the corresponding authors on reasonable request.

## Supplementary Materials

Supplementary Materials Calculation section Supplementary figures Fig. S1. The yield rate of CH_4_, C_2_H_4_, C_2_H_6_, and C_2_H_2_ at different times under 700 mJ per pulse of laser energy. Fig. S2. GC chromatogram of H_2_ and CO released from laser-induced CH_3_OH. Fig. S3. Gibbs free energy of the formation of ions and radicals in LBL-promoted conversion of CH_3_OH. Fig. S4. Mechanism for the formation of C_2_H_6_, C_2_H_4_, C_2_H_2_, and CH_4_ in CH_3_OH decomposition reaction under pulsed laser. Fig. S5. Carbon–carbon radial distribution functions. Fig. S6. Each step equations of CH_3_OH decomposition under pulsed laser. Fig. S7. Mechanism and free energy profiles for CO and H_2_ conversion reaction. Fig. S8. Half-life along with reaction temperature for the formation of CHOH step in CO and H_2_ conversion reaction. Fig. S9. Hydrogen−hydrogen radial distribution functions of the cooling stage. Fig. S10. Carbon–oxygen radial distribution functions of the cooling stage. Fig. S11. Carbon–carbon radial distribution functions of the cooling stage. Fig. S12. Key structures for CH_3_OH decomposition under pulsed laser.
