# Supplementary material for: Laser-Induced Methanol Decomposition for Ultrafast Hydrogen Production
Source: Research (Wash D C). 2023 May 9;6:0132. doi: 10.34133/research.0132 (PMC10204739; doi:10.34133/research.0132)
Supplement: Supplementary Materials — Calculation section Supplementary figures Fig. S1. The yield rate of CH4, C2H4, C2H6, and C2H2 at different times under 700 mJ per pulse of laser energy. Fig. S2. GC chromatogram of H2 and CO released from laser-induced CH3OH. Fig. S3. Gibbs free energy of the formation of ions and radicals in LBL-promoted conversion of CH3OH. Fig. S4. Mechanism for the formation of C2H6, C2H4, C2H2, and CH4 in CH3OH decomposition reaction under pulsed laser. Fig. S5. Carbon–carbon radial distribution functions. Fig. S6. Each step equations of CH3OH decomposition under pulsed laser. Fig. S7. Mechanism and free energy profiles for CO and H2 conversion reaction. Fig. S8. Half-life along with reaction temperature for the formation of CHOH step in CO and H2 conversion reaction. Fig. S9. Hydrogen−hydrogen radial distribution functions of the cooling stage. Fig. S10. Carbon–oxygen radial distribution functions of the cooling stage. Fig. S11. Carbon–carbon radial distribution functions of the cooling stage. Fig. S12. Key structures for CH3OH decomposition under pulsed laser. [file research.0132.f1.docx]

**Supplementary Information for**

**Laser-induced methanol decomposition for ultrafast hydrogen production**

Weiwei Cao ^a, b^†, Yinwu Li ^b^†, Bo Yan ^a, b^†, Zhiping Zeng ^a b^, Pu Liu ^a, b^,

Zhuofeng Ke ^b^* & Guowei Yang ^a, b^*

*^a^ State Key Laboratory of Optoelectronic Materials and Technologies, Nanotechnology Research Center,* *Sun Yat-sen University, Guangzhou 510275, P. R. China*

*^b^ School of Materials Science & Engineering, Sun Yat-sen University, Guangzhou 510275, P. R. China*

† These authors contributed equally to this work.

* Corresponding authors: [kezhf3@mail.sysu.edu.cn](mailto:kezhf3@mail.sysu.edu.cn), [stsygw@mail.sysu.edu.cn](mailto:stsygw@mail.sysu.edu.cn)

**Computational details**

The calculations of mechanism study were performed using Gaussian 16 program package,^1^ by the density functional theory (DFT) .^2,3^ Based on our experiment, the laser-induced bubble reactions were carried out in vacuoles condition. Hence, optimizations were taken in the gas phase for all the structures, with the ideal gas conditions at 0 K, at the M06-2X^4^ /def2-QZVP^5,6^ level of theory. All the 3D optimized structures were displayed by CYLview visualization program.^7^ In order to identify the structures to be minima or transition states, the analytical frequency calculations were performed at the same level of theory (M06-2X/ def2-QZVP). Intrinsic reaction coordinate (IRC) calculations were carried out, aiming to confirm the connection of a transition state and two correct minima. Basde on eq S1, all the Gibbs free energy were corrected by reaction temperature.

$G=\varepsilon_{0}+ H_{corrt}-TS_{tot}$ eq S1

In eq S1, the $\varepsilon_{0}$ is the total electronic energy. The $H_{corrt}$ is the thermal correction to enthalpy. The $S_{tot}$ is the total entropy. The reaction temperature is 10000 K in CH_3_OH decomposition reaction. For H_2_ and CO conversion, the reaction temperature is 298.15 K. Besides, the reaction rate is calculated based on Eyring’s transition state theory eq S2.

$k^{TST}= \sigma\frac{k_{B}T}{h}{(\frac{RT}{P_{0}})}^{\Delta n}e^{-\Delta G^{0,\neq}/(k_{B}T)}$ eq S2

In eq S2, the rotational symmetry number *σ* = 1. The *k_B_* is the Boltzmann constant. The *h* is the Planck constant. For the H_2_ and CO conversion reaction, the Δ*n* are 1 in the rate determining step. The half-life of the two reactions are based on eq S3. The reactants concentration are assumed to be 1M in the two reactions.

$t_{1/2}= \frac{1}{k^{TST}}$ eq S3

In order to investigate the reaction process, the *ab initio* MD simulations were performed using the CP2K program,^8^ at GFNE-xTB level of theory.^9,10^ The CP2K input files were generated with help of Multiwfn program.^11^ We performed MD simulations under periodic boundary conditions for 50 methanol molecules. The initial configurations for molecular dynamics simulations of methanol were built by using Packmol program.^12^ In order to conform to the thermochemical process with different temperatures, the MD simulation is carried out in NVT ensemble for 10 picosecond (ps), at 298 K, 5000 K and 10000 K, respectively. The cooling stage of the MD simulation used the annealing key word. The annealing coefficient is 0.999.

**References**

1 Gaussian 16, Revision C.01, M. J. Frisch, G. W. Trucks, H. B. Schlegel, G. E. Scuseria, M. A. Robb, J. R. Cheeseman, G. Scalmani, V. Barone, G. A. Petersson, H. Nakatsuji, X. Li, M. Caricato, A. V. Marenich, J. Bloino, B. G. Janesko, R. Gomperts, B. Mennucci, H. P. Hratchian, J. V. Ortiz, A. F. Izmaylov, J. L. Sonnenberg, D. Williams-Young, F. Ding, F. Lipparini, F. Egidi, J. Goings, B. Peng, A. Petrone, T. Henderson, D. Ranasinghe, V. G. Zakrzewski, J. Gao, N. Rega, G. Zheng, W. Liang, M. Hada, M. Ehara, K. Toyota, R. Fukuda, J. Hasegawa, M. Ishida, T. Nakajima, Y. Honda, O. Kitao, H. Nakai, T. Vreven, K. Throssell, J. A. Montgomery, Jr., J. E. Peralta, F. Ogliaro, M. J. Bearpark, J. J. Heyd, E. N. Brothers, K. N. Kudin, V. N. Staroverov, T. A. Keith, R. Kobayashi, J. Normand, K. Raghavachari, A. P. Rendell, J. C. Burant, S. S. Iyengar, J. Tomasi, M. Cossi, J. M. Millam, M. Klene, C. Adamo, R. Cammi, J. W. Ochterski, R. L. Martin, K. Morokuma, O. Farkas, J. B. Foresman, and D. J. Fox, Gaussian, Inc., Wallingford CT, (2016).

2 Kohn, W. & Sham, L. J. Self-Consistent Equations Including Exchange and Correlation Effects. *Phys. Rev*. 140, A1133-A1138, (1965).

3 Hohenberg, P. & Kohn, W. Inhomogeneous Electron Gas. *Phys. Rev*. 136, B864-B871, (1964).

4 Zhao, Y. & Truhlar, D. G. The M06 suite of density functionals for main group thermochemistry, thermochemical kinetics, noncovalent interactions, excited states, and transition elements: two new functionals and systematic testing of four M06-class functionals and 12 other functionals. *Theor. Chem. Acc.* 120, 215-241, (2007).

5 Weigend, F. Accurate Coulomb-fitting basis sets for H to Rn. *Phys. Chem. Chem. Phys.* 8, 1057-1065, (2006).

6 Weigend, F. & Ahlrichs, R. Balanced basis sets of split valence, triple zeta valence and quadruple zeta valence quality for H to Rn: Design and assessment of accuracy. *Phys. Chem. Chem. Phys.* 7, 3297-3305, (2005).

7 Legault, C. Y., CYLview, 1.0b; Université de Sherbrooke. (2009), <http://www.cylview.org>.

8 Kuehne, T. D. et al. CP2K: An electronic structure and molecular dynamics software package - Quickstep: Efficient and accurate electronic structure calculations. *J. Chem. Phys.* 152, 194103, (2020).

9 Bannwarth, C., Ehlert, S. & Grimme, S. GFN2-xTB-An Accurate and Broadly Parametrized Self-Consistent Tight-Binding Quantum Chemical Method with Multipole Electrostatics and Density-Dependent Dispersion Contributions. *J.* *Chem. Theory. Comput.* 15, 1652-1671, (2019).

10 Grimme, S., Bannwarth, C. & Shushkov, P. A Robust and Accurate Tight-Binding Quantum Chemical Method for Structures, Vibrational Frequencies, and Noncovalent Interactions of Large Molecular Systems Parametrized for All spd-Block Elements (Z=1-86). *J. Chem. Theory Comput.* 13, 1989-2009, (2017).

11 Lu, T. & Chen, F. Multiwfn: A multifunctional wavefunction analyzer. *J. Comput. Chem.* 33, 580-592, (2012).

12 Martinez, L., Andrade, R., Birgin, E. G. & Martinez, J. M. PACKMOL: A Package for Building Initial Configurations for Molecular Dynamics Simulations. *J. Comput. Chem.* 30, 2157-2164, (2009).





**Figure S1.** The yield rate of CH_4_, C_2_H_4_, C_2_H_6_, and C_2_H_2_ at different time under 700 mJ/pulse laser energy.


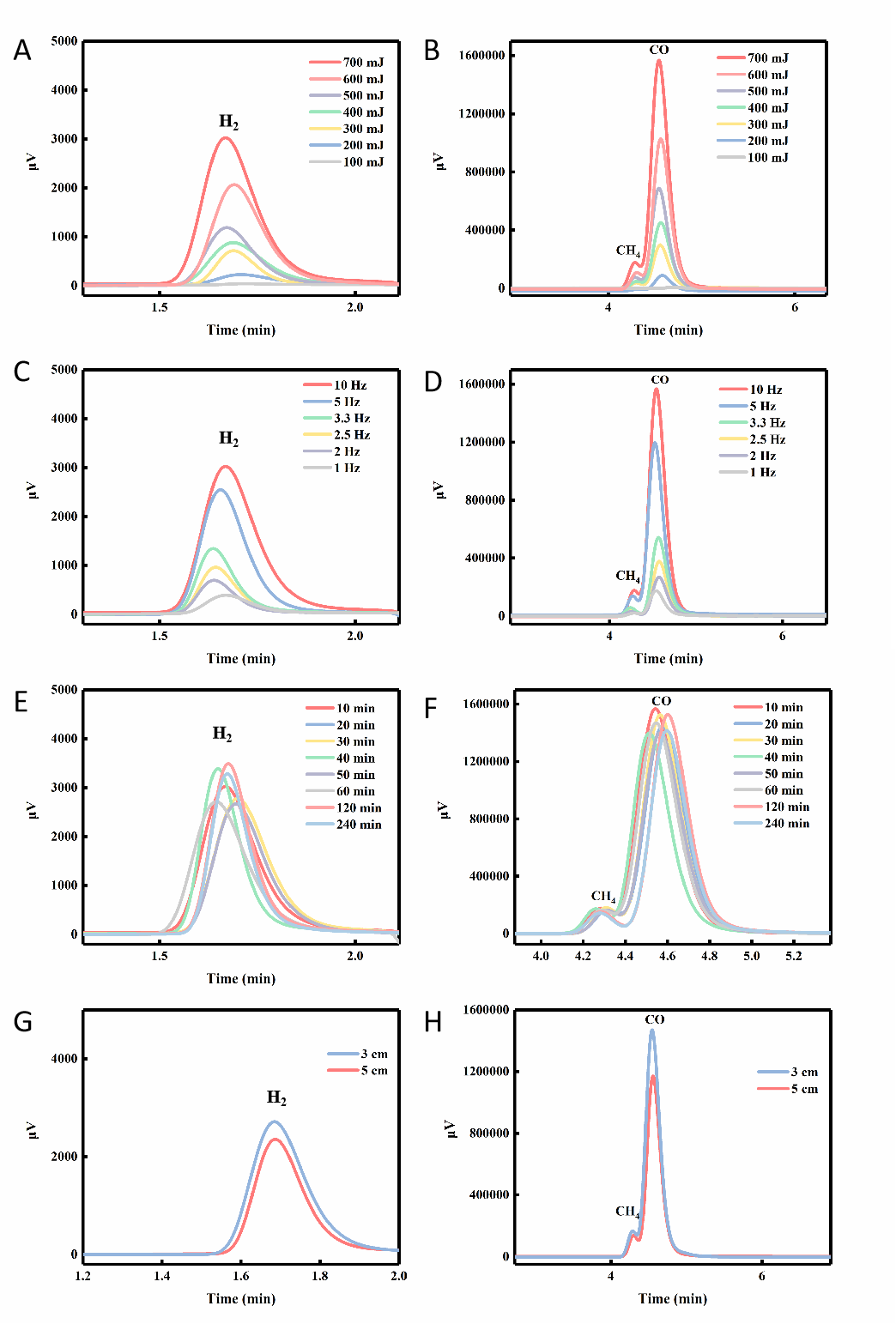


**Figure S2**. GC chromatogram of H_2_ and CO released from laser-induced CH_3_OH. (A, B) GC chromatogram of H_2_ and CO at different energy. (C, D) GC chromatogram of H_2_ and CO under different laser frequency. (E, F) GC chromatogram of H_2_ and CO under different laser time. (G, H) Gas chromatogram of H_2_ and CO released by laser focusing at different distances from the liquid level. It is found that the H_2_ concentration decreases with the distance between the laser focus and the liquid level. This may be caused by the loss of laser energy when the laser passes through the liquid.


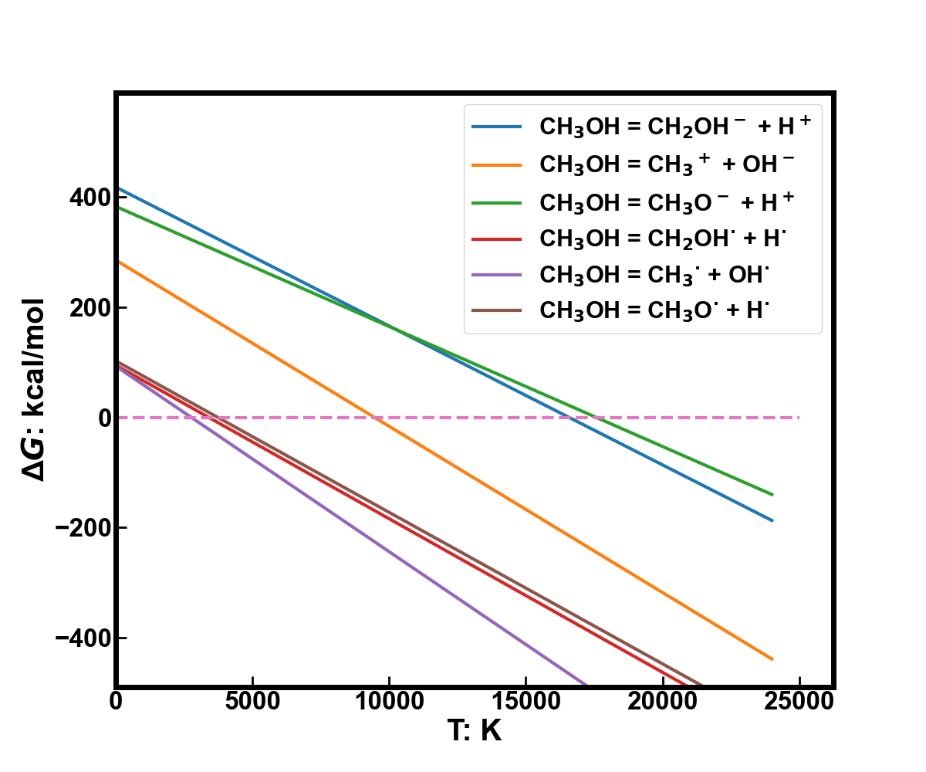


**Figure S3.** Gibbs free energy of the formation of ions and radicals in LBL-promoted conversion of CH_3_OH.





**Figure S4.** Mechanism for the formation of C_2_H_6_ C_2_H_4_ C_2_H_2_, and CH_4_ in CH_3_OH decomposition reaction under pulsed laser. The free energies calculated in 10000 K.





**Figure S5.** Carbon-Carbon radial distribution functions. The radial distribution functions are calculated with the 500 fs for each temperature.





**Figure S6.** Each step equations of CH_3_OH decomposition under pulsed laser. Energies of Δ*E* Δ*H* Δ*G* are shown in kcal/mol. Energy of Δ*S* is shown in cal/mol.

**Figure S7.** Mechanism and free energy profiles for CO and H_2_ conversion reaction. The free energies are calculated in 298.15K.


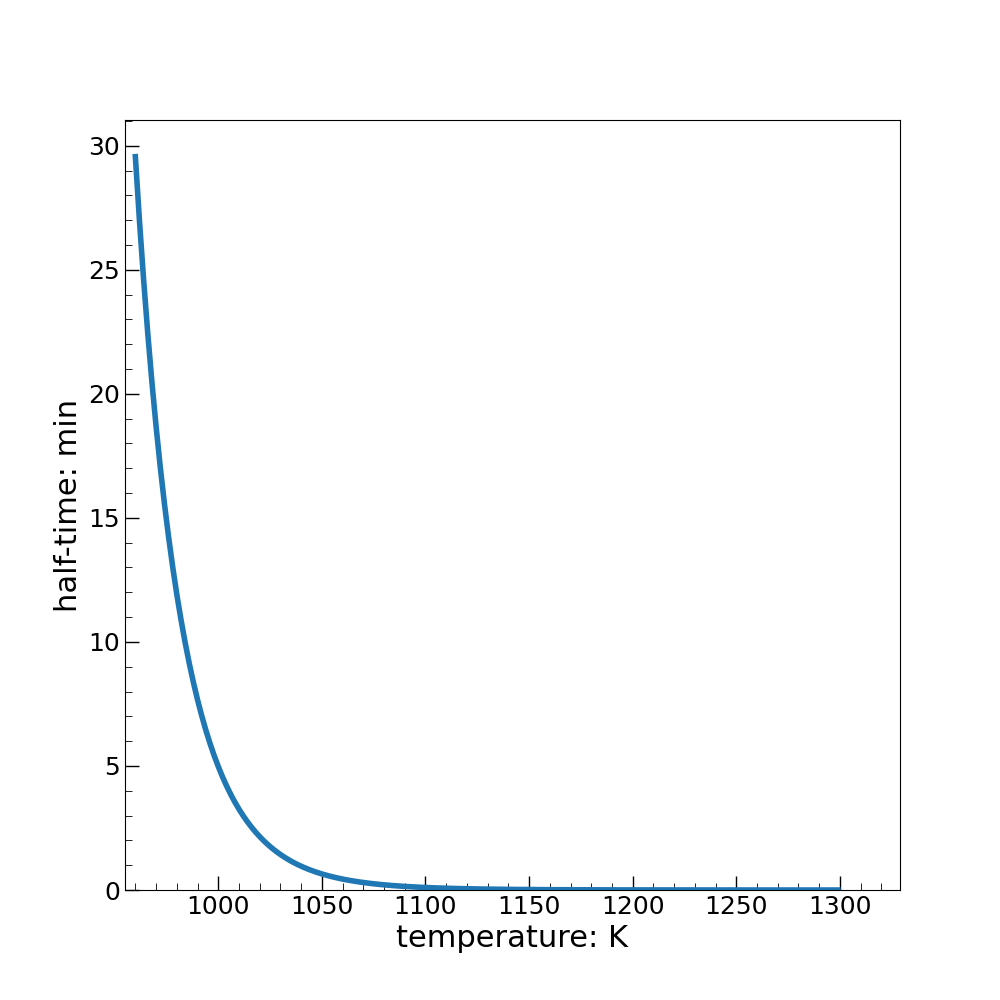


**Figure S8.** Half-life along with reaction temperature for the formation of CHOH step in CO and H_2_ conversion reaction.





**Figure S9.** Hydrogen−Hydrogen radial distribution functions of cooling stage. The radial distribution functions are calculated with the 500 fs for each temperature.





**Figure S10.** Carbon-Oxygen radial distribution functions of cooling stage. The radial distribution functions are calculated with the 500 fs for each temperature.





**Figure S11.** Carbon-Carbon radial distribution functions of cooling stage. The radial distribution functions are calculated with the 500 fs for each temperature.





**Figure S12.** Key structures for CH_3_OH decomposition under pulsed laser. The Bond distances are shown in Å.

**Cartesian coordinates (xyz) for all optimized structures**

H_2_

0 1

H 0.000000 0.000000 0.368931

H 0.000000 0.000000 -0.368931

O_2_

0 3

O 0.000000 0.000000 0.594019

O 0.000000 0.000000 -0.594019

CO

0 1

C 0.000000 0.000000 -0.639633

O 0.000000 0.000000 0.479725

C_2_H_2_

0 1

C 0.000000 0.000000 0.596506

H 0.000000 0.000000 1.658831

C 0.000000 0.000000 -0.596506

H 0.000000 0.000000 -1.658831

C_2_H_4_

0 1

C 0.000000 0.660642 0.000000

H 0.920892 1.226999 0.000000

H -0.920852 1.227040 -0.000000

C -0.000000 -0.660642 -0.000000

H -0.920892 -1.226999 -0.000000

H 0.920852 -1.227040 -0.000000

C_2_H_6_

0 1

C -0.000000 -0.000000 0.761768

H -0.000000 1.014493 1.156547

H 0.878577 -0.507246 1.156547

H -0.878577 -0.507246 1.156547

C -0.000000 0.000000 -0.761768

H 0.878577 0.507246 -1.156547

H -0.000000 -1.014493 -1.156547

H -0.878577 0.507246 -1.156547

CH_2_CH^•^

0 2

C 0.049494 -0.583293 0.000000

H -0.876767 -1.154029 0.000000

H 0.969712 -1.155642 0.000000

C 0.049494 0.717675 0.000000

H -0.686879 1.503377 0.000000

CH_2_O

0 1

C -0.000006 0.524599 0.000000

H -0.937618 1.104758 -0.000000

H 0.937701 1.104583 0.000000

O -0.000006 -0.669617 -0.000000

CH_2_OH^•^

0 2

C -0.680699 0.027973 0.064165

H -1.111881 0.990210 -0.166212

H -1.232880 -0.880047 -0.104651

O 0.666001 -0.125395 -0.021233

H 1.100947 0.725156 0.055736

CH_2_OH^-^

-1 1

C -0.766082 -0.000046 -0.154671

H -1.100642 0.876907 0.440430

H -1.100937 -0.876494 0.441018

O 0.724872 -0.000055 -0.097008

H 0.999090 0.000301 0.822642

CH_3_^•^

0 2

C -0.000000 -0.000000 -0.000245

H 0.000000 1.075560 0.000490

H -0.931462 -0.537780 0.000490

H 0.931462 -0.537780 0.000490

CH_3_^+^

1 1

C 0.000000 -0.000000 -0.000013

H -0.000000 1.088740 0.000025

H -0.942877 -0.544370 0.000025

H 0.942877 -0.544370 0.000025

CH_3_CH

0 1

C -0.591724 0.022342 -0.031516

H -1.022194 0.965162 -0.374543

H -0.582591 0.070975 1.088455

C 0.842283 -0.190160 -0.041149

H -1.204340 -0.839182 -0.282948

H 1.305767 0.809949 0.005028

CH_3_CH_2_^•^

0 2

C -0.011506 -0.691776 -0.000000

H 1.006148 -1.099775 -0.000000

H -0.507447 -1.093769 -0.882318

H -0.507447 -1.093769 0.882318

C -0.011506 0.792036 0.000000

H 0.073406 1.342878 -0.922542

H 0.073406 1.342878 0.922542

CH_3_O^•^

0 2

C -0.575740 0.000545 0.012044

H -0.998673 0.911377 0.444662

H -0.995846 -0.896877 0.475106

H -0.870945 -0.019829 -1.049110

O 0.789988 0.000258 0.007135

CH_3_O^-^

-1 1

C 0.000000 -0.000000 -0.539521

H 0.000000 1.018945 -1.030351

H -0.882433 -0.509473 -1.030351

H 0.882433 -0.509473 -1.030351

O -0.000000 0.000000 0.791022

CH_3_OH

0 1

C 0.661914 -0.020516 0.000000

H 1.082700 0.981153 -0.000001

H 1.019802 -0.544140 -0.888729

H 1.019802 -0.544138 0.888730

O -0.743527 0.122108 0.000000

H -1.145573 -0.746643 0.000000

CH_4_

0 1

C 0.000000 0.000000 0.000000

H 0.626947 0.626947 0.626947

H -0.626947 -0.626947 0.626947

H -0.626947 0.626947 -0.626947

H 0.626947 -0.626947 -0.626947

CHO^•^

0 2

C 0.061556 0.579700 -0.000000

H -0.861783 1.211878 0.000000

O 0.061556 -0.586259 0.000000

CHOH.log

0 1

C -0.731381 -0.153626 -0.000104

H -1.103367 0.898715 0.000377

O 0.567619 -0.096437 0.000088

H 0.950703 0.794541 -0.000461

TS_1_

0 1

C -0.514296 -0.163188 0.000064

H -1.024430 -0.497963 -0.912902

H -1.101545 1.107122 -0.000033

H -1.024272 -0.497747 0.913198

O 0.797624 -0.023629 -0.000067

H -0.144969 1.056745 -0.000118

TS_1_-iso

0 1

C -1.726960 0.336954 0.198038

H -2.341578 0.836051 -0.561780

H -0.724070 1.426060 0.177822

H -2.020847 0.582342 1.226485

O -1.202189 -0.784618 -0.050445

H 0.065905 1.062274 -0.201608

H 0.142397 -0.469249 -0.565168

O 0.925592 0.201500 -0.648529

C 1.858329 -0.066433 0.393911

H 1.345076 -0.195660 1.346700

H 2.411842 -0.971962 0.158875

H 2.545843 0.771964 0.458777

TS_2_

0 1

C 0.001024 0.658710 -0.000000

H 1.005577 1.123335 -0.000000

H -1.019906 -0.058382 0.000000

O 0.001024 -0.627152 0.000000

TS_2_-iso

0 1

C -1.468032 0.621181 0.036802

H -2.469758 0.993175 0.306994

O -1.376790 -0.623716 0.162100

H -0.250431 -0.711380 -0.199984

H -0.068480 0.743827 -0.465134

O 0.743483 -0.064693 -0.628367

C 1.760284 0.072366 0.344045

H 2.210172 -0.897986 0.545541

H 2.531156 0.747519 -0.022961

H 1.360276 0.470829 1.280600

TS_3_

0 1

C 0.598187 -0.322823 -0.000021

H 1.209694 0.891619 0.000166

O -0.617453 0.011065 0.000020

H 0.140808 0.956798 -0.000199

TS_3_-iso

0 1

C -1.845064 0.443278 0.316126

H -0.877317 1.392917 0.170735

O -1.374911 -0.610821 -0.051932

H 0.168551 1.013601 -0.305778

H -0.096572 -0.438385 -0.475939

O 0.789724 0.173747 -0.634637

C 1.758805 -0.102832 0.386094

H 2.531688 0.656637 0.332126

H 1.288157 -0.089637 1.367317

H 2.184540 -1.081212 0.190774

A-TS_1_

0 1

C 0.608748 0.000001 -0.189385

H 1.439483 -0.000005 1.135220

H 1.091057 0.909891 -0.513075

H 1.091058 -0.909886 -0.513083

C -0.817075 0.000000 -0.034123

H -1.336782 0.930295 0.110476

H -1.336783 -0.930296 0.110470

H 0.301930 -0.000004 1.011040

A-TS_2_

0 1

C -0.596003 0.038539 -0.046730

H -1.086156 1.013677 -0.062274

H -0.103918 -0.201795 1.019516

C 0.793374 -0.174098 -0.088657

H -1.264162 -0.806864 -0.188335

H 1.270010 0.808338 0.043416

A-TS_3_

0 1

C -0.428135 0.220034 0.000002

H -1.094785 1.066518 0.000014

H -1.298567 -0.766136 0.419969

C 0.784130 -0.231644 0.000012

H -1.298517 -0.766099 -0.420132

H 1.555895 0.535380 0.000069
